# Deletion and Randomization of Structurally Variable Regions in *B. subtilis* Lipase A (BSLA) Alter Its Stability and Hydrolytic Performance Against Long Chain Fatty Acid Esters

**DOI:** 10.3390/ijms21061990

**Published:** 2020-03-14

**Authors:** Ronny Martínez, Claudia Bernal, Rodrigo Álvarez, Christopher Concha, Fernando Araya, Ricardo Cabrera, Gaurao V. Dhoke, Mehdi D. Davari

**Affiliations:** 1 Departamento de Ingeniería en Alimentos, Instituto de Investigación Multidisciplinaria en Ciencia y Tecnología, Universidad de La Serena, Av. Raúl Bitrán 1305, La Serena 1720010, Chile; cbernal@userena.cl (C.B.); rialvarez@userena.cl (R.Á.); christopherconchapujado@gmail.com (C.C.); 2 Escuela de Tecnología Médica, Facultad de Salud, Sede La Serena, Universidad Santo Tomás, La Serena 1710172, Chile; 3 Laboratorio de Bioquímica y Biología Molecular, Departamento de Biología, Facultad de Ciencias, Universidad de Chile, Santiago 7800003, Chile; fernando.araya.p@ug.uchile.cl (F.A.); ricabrer@uchile.cl (R.C.); 4 Lehrstuhl für Biotechnologie, RWTH Aachen University, Worringerweg 3, 52074 Aachen, Germany; g.dhoke@biotec.rwth-aachen.de (G.V.D.); m.davari@biotec.rwth-aachen.de (M.D.D.)

**Keywords:** enzyme engineering, loop deletion, structure-function, BSLA, lipase

## Abstract

The continuous search for novel enzyme backbones and the engineering of already well studied enzymes for biotechnological applications has become an increasing challenge, especially by the increasing potential diversity space provided by directed enzyme evolution approaches and the demands of experimental data generated by rational design of enzymes. In this work, we propose a semi-rational mutational strategy focused on introducing diversity in structurally variable regions in enzymes. The identified sequences are subjected to a progressive deletion of two amino acids and the joining residues are subjected to saturation mutagenesis using NNK degenerate codons. This strategy offers a novel library diversity approach while simultaneously decreasing enzyme size in the variable regions. In this way, we intend to identify and reduce variable regions found in enzymes, probably resulting from neutral drift evolution, and simultaneously studying the functional effect of said regions. This strategy was applied to *Bacillus. subtilis* lipase A (BSLA), by selecting and deleting six variable enzyme regions (named regions 1 to 6) by the deletion of two amino acids and additionally randomizing the joining amino acid residues. After screening, no active variants were found in libraries 1% and 4%, 15% active variants were found in libraries 2% and 3%, and 25% for libraries 5 and 6 (n = 3000 per library, activity detected using tributyrin agar plates). Active variants were assessed for activity in microtiter plate assay (*p*NP-butyrate), thermal stability, substrate preference (*p*NP-butyrate, -palmitate), and compared to wildtype BSLA. From these analyses, variant P5F3 (F41L-ΔW42-ΔD43-K44P), from library 3 was identified, showing increased activity towards longer chain *p*-nitrophenyl fatty acid esters, when compared to BSLA. This study allowed to propose the targeted region 3 (positions 40–46) as a potential modulator for substrate specificity (fatty acid chain length) in BSLA, which can be further studied to increase its substrate spectrum and selectivity. Additionally, this variant showed a decreased thermal resistance but interestingly, higher isopropanol and Triton X-100 resistance. This deletion-randomization strategy could help to expand and explore sequence diversity, even in already well studied and characterized enzyme backbones such as BSLA. In addition, this strategy can contribute to investigate and identify important non-conserved regions in classic and novel enzymes, as well as generating novel biocatalysts with increased performance in specific processes, such as enzyme immobilization.

## 1. Introduction:

Proteins are the functional and structural units in biological systems. Among proteins, enzymes have evolved over millions of years to specifically carry out essential functions in living organisms [1,2]. Due to their catalytic properties, enzymes are currently used in industrial and consumer processes [3]. Enzymes have several advantages over non-biological catalysts and synthetic reagents, which often require extreme reaction conditions and can generate undesired side- or mixed enantiomeric products [4]. Enzyme biocatalysts on the other hand, can perform specific chemical reactions in mild aqueous conditions, requiring less energy input and producing less, and more manageable, waste [5]. As a result, the demand for enzymes fit for industrial and consumer purposes has increased steadily over the last 50 years. On the other hand, enzyme operating conditions are quite narrow; enzymes are generally limited to physiological conditions of their source organism and are highly sensitive to changes in such parameters [6].

However, as immense as natural diversity is, reaction conditions, reaction chemistry, as well as substrate and product concentrations in which industrial processes are conceived often differ from physiological conditions, or combine specific conditions that do not simultaneously occur in nature, resulting in reduced enzymatic efficiency, stability, or even complete loss of catalytic activity [1].

As a solution to this challenge, enzymes can be artificially evolved or engineered to increase their performance under conditions very rarely found as simultaneous selective pressure in nature, such as high activity at low temperatures and high thermal stability [6,7] or in the presence of organic solvents [8]. Understanding sequence, structural, and functional factors that govern enzyme adaptation will allow us to quickly and efficiently engineer enzymes for specific applications, tailoring them for specific downstream processing, storage, and final activity conditions [9].

Candidate enzymes can be optimized through enzyme engineering [10], enzyme immobilization, or as recently proposed, a combination of both [11]. Successfully engineered enzymes for non-natural conditions enabled biocatalysts to be efficiently used in industrial processes which were traditionally performed chemically. As a result, the global market for industrial enzymes has grown from 2.9 billion USD in 2008 to a projected 6.2 billion USD in 2020 [12].

Enzyme engineering strategies seek to generate suitable enzymes with a higher performance compared to the starting variant [1] using strategies ranging from iterative screening of randomly mutated variants for the development of a specific property to the rational modification or design of the catalytic site of the target enzyme for improved or new biocatalytic activities.

The most employed strategy in enzyme engineering is directed evolution [13]. Through iterative cycles of gene diversity generation and high throughput screening, improved variants from a parent can be generated and identified. The recent recognition of the field through the Nobel Prize in Chemistry awarded to Prof. Frances H. Arnold and the work of many other renown researchers to develop the field [14,15,16,17] have shown that directed evolution is a robust enzyme engineering strategy and in addition can provide valuable insights in structure–function relationships in well-known and recently isolated and less studied enzymes [18,19]. Still, directed evolution as a strategy faces many challenges; most strategies involve making relatively small changes to existing enzymes by introducing amino acid diversity either randomly or targeted based on a rational hypothesis [2]. Therefore, protein characteristics such as size, length, and structural features are not dramatically modified in a single round using these approaches.

Through rational and semi-rational protein engineering approaches, researchers can target specific enzyme residues or residues based on in silico, structural, or computational chemistry approaches. These strategies are particularly effective at improving catalytic properties of the enzymes such as substrate specificity, stereo- and regioselectivity, and generating new catalytic activities. Most of these rational approaches also focus on exchanging amino acids, as in directed evolution approaches, and the potential variation of the overall sequence and structure size of a protein is largely not addressed. However, the use of protein sequence and structure to bring targeted variability to the backbone of enzymes is an incipient idea as a potential new protein engineering strategy [20,21,22].

Changes in sequence length are observed in a limited number of recombination methods and very specific rational approaches [2,23]. With a fixed enzyme length and the successful development of efficient enzyme engineering approaches, certain specific enzyme backbones—such as subtilisin protease or phytase folds—have seen close to all positions mutated and either reported or patented [24]. In this scenario, academic and industry researchers are eager to discover new backbones of classical enzyme classes for potential new knowledge or products [25].

A wide variety of interesting enzymes backbones, especially from extreme environments, have been identified [26,27]. However, wild-type enzymes found in nature are no longer directly applied in industrial processes. In fact, novel enzymes are greatly benefited from a single iteration of directed evolution or enzyme engineering [2,28]. Currently, enzyme discovery is focused on finding new backbones for generally applied enzyme classes, followed by enzyme engineering in order to bring novel enzymes up to industry standards, especially in terms of specific activity and stability. At this point, backbone variability is still provided by nature, whereas protein engineering approaches are providing more than enough sequence variability to generate libraries with great diversity. Our question is, how can we start providing our own (non-natural) backbone and sequence variability without generating an unmanageable diversity explosion?

Most enzyme engineering approaches still struggle with the inherent diversity generated by amino acid exchanges in a given protein sequence, studies reporting changes in protein sequence length are relatively infrequent, mostly describing binding domain, N- or C-terminal deletion [20,29]. Studies on introducing size variation to a protein backbone while optimizing it are presenting alternative solutions with a few reports pushing to validate the idea [21,22,30,31]. There are several proposed advantages for decreasing the size of an enzyme, especially on solvent exposed and loop regions [30]. With current screening limitations on most research laboratories, there is not much sense in simultaneously randomizing sequence and size of a protein. To achieve library sizes which are feasible to fully screen, a rational component must be added in order to limit and focus the size change and randomization on specific regions of the protein structure.

It has been proposed that protein engineering strategies must also “evolve” to avoid getting trapped on inherent protein characteristics coming from their natural evolution path [9]. Among possible evolution paths, we believe that pushing enzymes to a different (reduced) size is a not a typical natural evolutive pressure and therefore enzyme backbones provided by nature are probably not optimized for size, with the exception—perhaps—of secreted enzymes.

**Figure 1:**
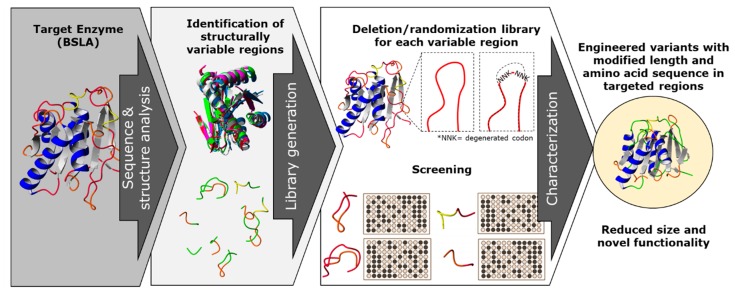
A combination of rational and random mutagenesis approaches is proposed to achieve targeted and simultaneous variation on protein sequence length and amino acid residues. With the premise that reducing the sequence size of enzymes leads to a “overall size optimization” process, we aim to identify and target structurally non-conserved regions of the protein (variable in sequence and size) for a deletion/randomization process. The targeted sequences are subjected to deletion of two amino acid residues, and the new junction will be randomized as an NNK codon for each joining residue in order to allow the new junction to find a new stable amino acid pair, generating a double site saturation mutagenesis library for each targeted region. This strategy offers a unique combination of sequence deletion and randomization by generating a novel type of diversity to be screened and generating enzyme variants which have probably not yet been described.

In this work, we propose a combination of rational and random mutagenesis approaches to achieve targeted variation on the protein sequence length, with the advantage of increased chances of finding active variants (Figure 1). With the premise that pushing for altering the sequence size of enzymes could lead to a parallel optimization process, we aim to target structurally non-conserved regions of the protein (variable in sequence and size) for a deletion/randomization process. The sequence of a target region in the enzyme was selected and two residues were deleted, and the new junction was randomized using an NNK codon for each joining residue in order to allow the new junction to find a new stable amino acid pair. This strategy offers a unique combination of sequence deletion and randomization by generating a novel type of diversity to be screened.

We applied our strategy to *B. subtilis* lipase A (BSLA), a 180 amino acid lipase which has been used as model for solvent [32,33,34,35], thermal stability studies [36,37], and low temperature activity [38], and all of its amino acid positions have been subjected to site saturation mutagenesis [32,39]. Thus, BSLA serves an example of a highly studied enzyme backbone, with lower chance of finding novel, non-previously described variants with modified enzymatic or stability performance. Additionally, BSLA expression systems are described and enzymatic assays have been developed for screening and characterization [40,41]. We selected six structurally variable regions of the enzyme and subjected each of them to the proposed diversity generation strategy. The resulting libraries were screened for esterolytic activity using a butyrate ester chromogenic substrate and compared to the activity of the parental BSLA enzyme. The identified active variants for each library were also compared to wildtype regarding isopropanol resistance, hydrolysis of longer aliphatic chain substrates, and thermal resistance.

## 2. Results and Discussion

The results of this work are presented as the design and construction of the deletion/randomization libraries, variant screening for each library, variant characterization and comparison against wildtype (WT) BSLA, and structure–function analysis of the most interesting variant P5F3.

### 2.1. Identification and Selection of Structurally Variable Regions for BSLA

**Figure 2:**
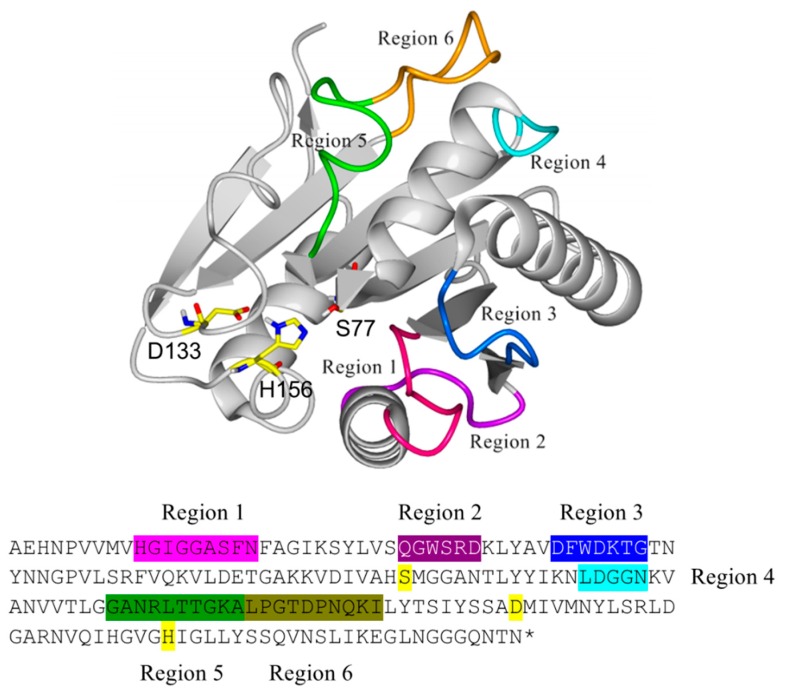
Structural location of the regions in *Bacillus subtilis* lipase A (BSLA) targeted for deletion/randomization and its equivalent location in the primary sequence with matching colors. The catalytic triad (Ser 77, Asp 133, and His 156) is highlighted in yellow.

The structure of BSLA (PDB 1I6W, chain A) was aligned with other 25 structures of prokaryote lipases using the YASARA software [42], MUSTANG routine, as well as with 30 PDB neighbors using the FatCat routine. The resulting alignment allowed to discriminate residues of the conserved α/β hydrolase motif and those corresponding to variable structures across the aligned structures. For BSLA, six regions were identified based on a high root mean square deviation (RMSD) with the structurally aligned enzymes, which were targeted for the generation of six deletion/randomization libraries (Figure 2). Despite being also loop (coil) structures, the regions 130–140 and 153–162 were structurally highly conserved, most likely because they contain the catalytic residues Asp 133 and His 156 and therefore their structural configuration is required, together with Ser 77, for catalysis.

### 2.2. Deletion/Randomization Strategy

Since the strategy aims to decrease the size and randomize the variable region, the deletion was designed to start in the “middle” of the sequence, whereby the neighboring “joining” amino acid are randomized each with a NNK codon, allowing for any amino acid combination (out of 400) to be obtained. The deletion of the identified regions through structurally alignments was performed from the middle of the selected sequence because the targeted sequences were mostly loop regions, and the amino acid residues from the “middle” of the loop show less interaction with the “core” backbone of the enzyme, suggesting that their deletion should have less impact on the general enzyme structure, compared to the “extreme” residues of the loop regions. The new neighbor residues were randomized to allow for the identification of a compatible amino acid residue pair in each deletion library assuming that the amino acid pair resulting from only the deletion most probably would generate an inactive variant due to (steric) incompatibility rather than sequence length. Additionally, two amino acid residues were deleted, because a single amino acid deletion would force a downstream change in spatial amino acid orientation, which we considered potentially too disturbing for a small 181 amino acid enzyme backbone (Table 1). Thus, a two amino acid deletion would keep the residue orientation of the original enzyme. Sampling this amino acid combination would allow to identify those compatible combinations that would result in an active enzyme—if possible—for each new loop length. BSLA was employed in this work to perform and assess this diversity strategy since it is a widely studied enzyme, it has a reduced size (181 aa) which decreases the number of potential libraries to be generated and increases the chance to generate variants with functional changes in hydrolytic performance. Of course, at the same time, by choosing a minimal, very optimized enzyme, we had the risk of finding only inactive variants after subjecting BSLA to the deletion/randomization strategy. Additionally, we aimed to generate variants which were not previously reported for a highly studied enzyme, supporting the potential of this diversity generation strategy to expand the sequence diversity on overstudied enzyme backbones, as this is that case for many industrially relevant enzymes.

### 2.3. Library Construction and Screening

The libraries (Lib 1 to Lib 6) were generated using a modified Quick change PCR [43], simultaneously introducing the deletion and two NNK degenerate codons using specific primers for each library (Table A1), the resulting libraries were transformed into *E. coli* BL21 Gold (DE3) cells for activity screening (≈3000 colonies each), the correct construction for each library was confirmed by DNA sequencing. Libraries were initially assessed in agar plates using tributyrin as a substrate for esterolytic activity. Overall, ≈18,000 colonies were picked from transformation plates to activity agar plates. Clones showing activity halo in tributyrin agar plates after 24 h were further assessed through a microtiter plate assay using *p*-nitrophenyl butyrate (*p*NPB) as a short chain fatty acid substrate. Lib 1 contained no variants forming a halo in activity agar plates, and ≈180 clones were picked for further sequence analysis. Lib 2, 15% showed some of halo formation many of them very faint, and ≈180 clones were picked into MTP plates. Lib 3 also contained 15% of halo forming colonies, however halos were clearer, resulting in ≈360 colonies picked to microtiter plates. Lib 4 contained no halo-forming variants, and ≈180 colonies were picked for further analysis. Lib 5 contained 25% variants with clear halos, and ≈450 colonies were picked to microtiter plates. Finally, Lib 6 also contained 25% of agar plate active variants, whereas ≈360 colonies were transferred to microtiter plates.

Microtiter plate assay (*p*NPB hydrolysis) screening revealed no active variants for library 1 and 4, whereas libraries 2 and 6 showed active variants with less than 40% the activity of wildtype. Libraries 3 and 5 contained variants with similar activity when compared to wildtype BSLA.

Active (when available) and inactive variants in tributyrin agar plates were sequenced for each library and summarized in Table 2. For libraries 3 and 5, a pattern for the active variants becomes apparent. For library 3 (positions 40–46), variants containing MG, LG, FP, and LP as new neighboring pairs were more abundant and more active, with less active variants FH, ME, and LM found. For library 5 (positions 104–113), 14 different active variants were identified having glycine as the second position amino acid in the new pair. Additional active variants (8) were identified having P, A, V, and S in the second position, suggesting probable steric limitation for the new length in these regions. In fact, 49% of all sequenced active variants contained glycine in the newly formed residue pairs, whereas only 11% of inactive variants contained a glycine, which is close to the random chance of having a glycine in an amino acid pair. If the prevalence of glycine in active variants could be used as an indicator for steric stress in the newly formed loops, Lib 2 and Lib 5 would show a higher need for small glycine residues for having an active configuration, whereas variants from Lib 3 and Lib 6 would still achieve active conformation with bulkier amino acid residues.

### 2.4. Active Variant Analysis

From all microtiter plate assay active variants, the five most active, P5F3 (Lib 3 LP, F41L-ΔW42-ΔD43-K44P), P5E4 (Lib 3 FP, F41-ΔW42-ΔD43-K44P), P11E4 (Lib 5 FG, R107F-ΔL108-ΔT109-T110G), P11C7 (Lib 5 GG, R107G-ΔL108-ΔT109-T110G), and P11F12 (Lib 5 YG, R107Y-ΔL108-ΔT109-T110G) were further assessed. Variants from Lib 2 and Lib 6 did not contain variants showing activity higher than 20% of wildtype BSLA.

**Figure 3:**
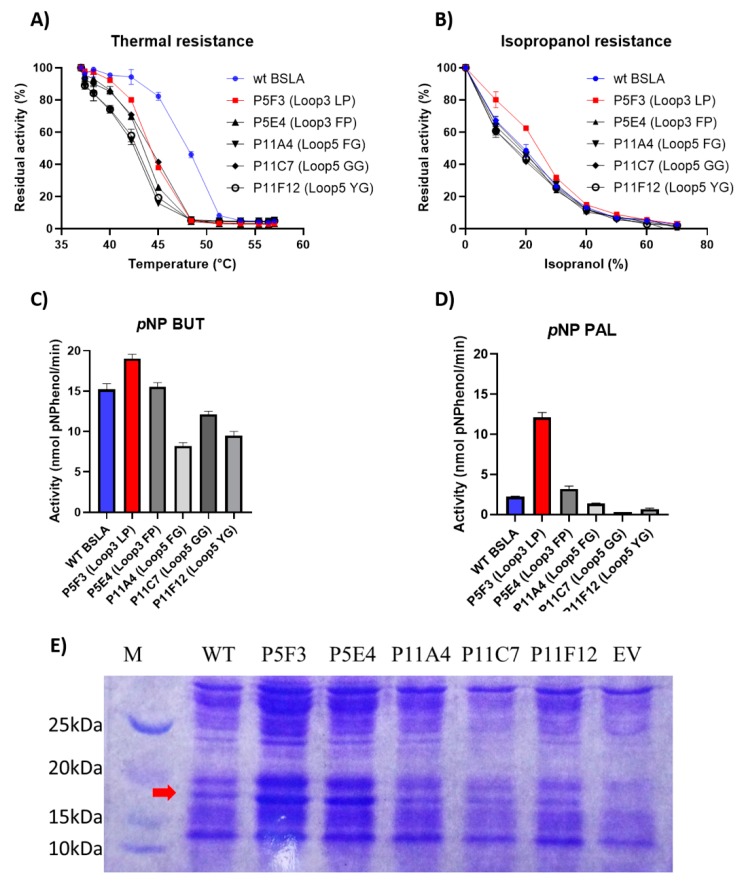
Comparison of the performance of WT BSLA and P5F3 (Lib 3 LP, F41L-ΔW42-ΔD43-K44P), P5E4 (Lib 3 FP, F41-ΔW42-ΔD43-K44P), P11E4 (Lib 5 FG, R107F-ΔL108-ΔT109-T110G), P11C7 (Lib 5 GG, R107G-ΔL108-ΔT109-T110G), and P11F12 (Lib 5 YG, R107Y-ΔL108-ΔT109-T110G) identified from the deletion/randomization libraries. Thermal resistance after 30 min incubation 37–57 °C. (**A**) Isopropanol resistance (0%–70%) (**B**), hydrolytic activity against *p*-nitrophenyl butyrate (**C**), and *p*-nitrophenyl palmitate (**D**) were assessed. SDS-PAGE analysis of the cell culture supernatant (**E**) revealed that enzyme production levels are different among the assessed variants, and thus enzyme amount should be standardized for further comparison. EV = empty vector. A complete image of the SDS-PAGE can be found in Figure A4.

Thermal resistance of the identified variants was lower when compared to wildtype BSLA, resulting in an inactivation curve displaced 4–5 °C to lower temperatures (Figure 3A). This result suggests that the changes introduced in Lib 3 and Lib 5 significantly affected the stability of the enzyme fold.

**Figure A1:**
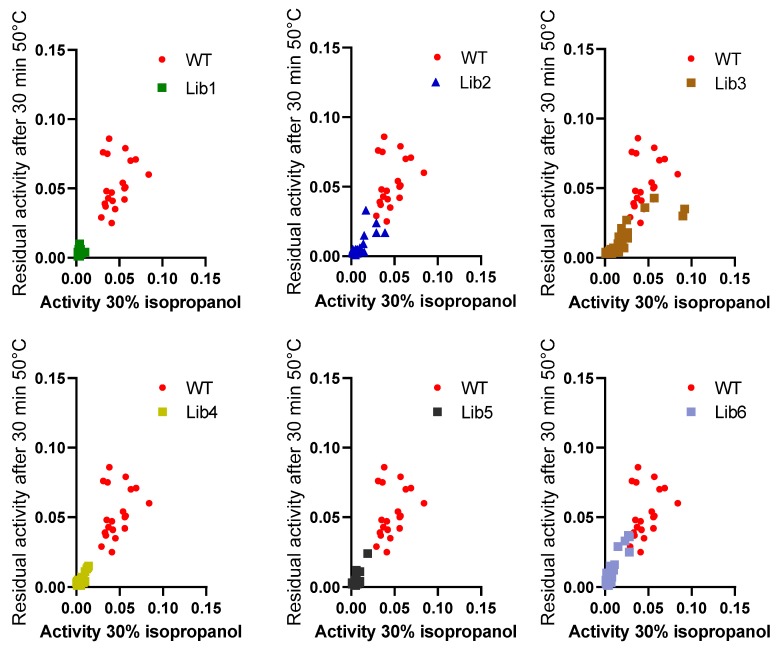
Variants from all libraries that were picked into microtiter plates (≈1700 clones) were screened for thermal resistance (52 °C for 30 min) and for *p*NPB esterolytic activity in the presence 30% isopropanol. No variants were identified with increased thermal resistance, and only variant P5F3 (F41L-ΔW42-ΔD43-K44P) was recovered from screening towards increased activity in the presence of isopropanol.

However, the resistance of the variants against increasing isopropanol content in the hydrolysis of *p*NPB was not changed when compared to wildtype BSLA. In fact, variant P5F3 shows a higher residual activity at 10% and 20% isopropanol (Figure 3B). The explanation for this increased performance, compared to thermal resistance, might be due to a modified substrate interaction or active site rearrangement. The results also point to different thermal and solvent inactivation mechanisms, since the difference in thermal resistance from wildtype BSLA and the deletion/randomization variants is clear, whereas the inactivation curve by isopropanol is not significantly different for all variants and wildtype BSLA, except for P5F3. All variants from Lib 1–6 that were picked for microtiter plate analysis (≈1700 clones) were screened for thermal resistance (52 °C for 30 min) and for *p*NPB esterolytic activity in the presence 30% isopropanol. No variants were identified with increased thermal resistance, and only variant P5F3 was recovered from screening for increased activity in the presence of isopropanol (Figure A1).

To further investigate possible changes in the hydrolytic performance of the variants, *p*-nitrophenol esters of butyrate (C4) and palmitate (C16) were used as substrates (Figure 3C,D). As previously reported for BSLA, the hydrolytic activity decreases for substrates with longer aliphatic chains. This is usually explained by the lack of a hydrophobic lid in BSLA, which is typical for lipases of this fold [44] and is described as a part of a water/oil interfacial activation mechanism. This decrease in activity, especially for wildtype BSLA could be due to a reduced substrate affinity resulting from the increasing length of the aliphatic chain, probably combined with a reduced substrate availability (or decreased effective substrate concentration due to low water solubility). On the other hand, variant P5F3 (Lib 3 LP) shows a different behavior, whereas hydrolytic activity does not dramatically decrease with substrates with a longer aliphatic chain. Interestingly, variant P5E4 (Lib 3 FP), shows a behavior similar to wildtype BSLA, suggesting that the length of region 3 is not the main explanation for the behavior of variant P5F3, pointing to the specific amino acid residues forming the new pair.

**Figure 4:**
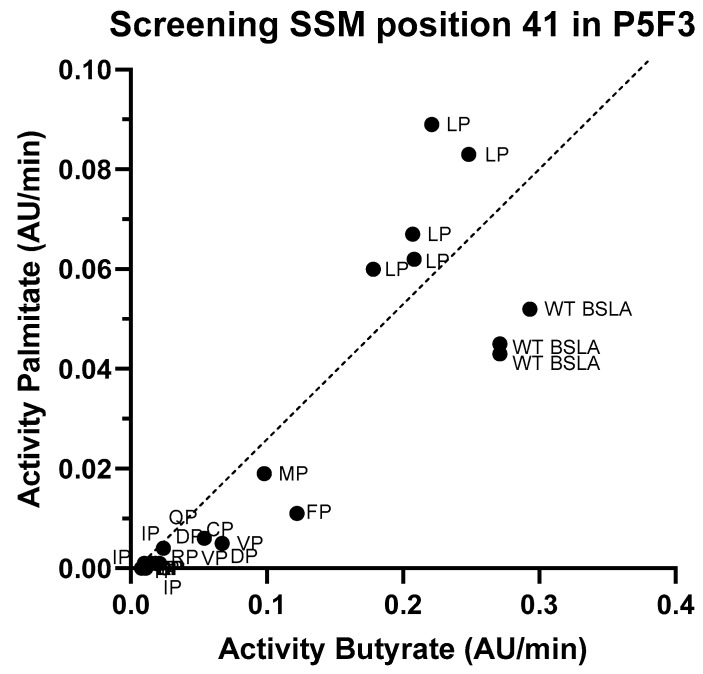
Screening results for the site saturation mutagenesis in position 41 on variant P5F3. After assessing hydrolytic activity against *p*-nitrophenyl butyrate and palmitate on the resulting library, and sequencing analysis of the best performing variants, results suggests that variant P5F3 (F41L-ΔW42-ΔD43-K44P) with the new LP amino acid pair in region 3 shows the highest activity against the longer fatty acid chain substrate.

Since the second position (44) of the new amino acid pair in both Lib 3 variants (P5F3 and P5E4) is proline, the effect of the first amino acid (position 41, originally phenylalanine) was studied by generating a site saturation mutagenesis library, and screening for *p*-nitrophenyl butyrate and palmitate hydrolysis (Figure 4). Activity screening and sequencing results showed that in fact the combination LP (F41L-ΔW42-ΔD43-K44P) showed the highest activity against *p*-nitrophenyl palmitate and a hydrolytic activity against *p*-nitrophenyl butyrate close to that of wildtype. Variants MP (F41M-ΔW42-ΔD43-K44P) and FP (F41-ΔW42-ΔD43-K44P) were also active, but showed significantly lower hydrolytic activity, whereas variants VP (F41V-ΔW42-ΔD43-K44P), CP (F41C-ΔW42-ΔD43-K44P) have very low overall activity (less than 10% of the original BSLA activity).

After screening six libraries of deletion and randomization of non-structurally conserved regions of BSLA, active variants with a supernatant activity close to the level of wildtype BSLA were identified in Lib 3 and Lib 5. Though variants from Lib 5 showed no improvement in the assessed parameters (hydrolytic activity, thermal, isopropanol resistance), variant P5F3 was identified from Lib 3, showing an increased hydrolytic activity against *p*-nitrophenyl palmitate when compared to wildtype BSLA, suggesting that region 3 and its deletion and double amino acid substitution could have an effect on the hydrolytic performance for substrates with different fatty acid chain lengths.

### 2.5. Characterization of Variant P5F3

To study and compare variant P5F3 against wildtype BSLA, an enzyme purification experiment was attempted. The cell culture supernatants containing the respective variants were concentrated using a 10 kDa mini crossflow unit and filtered through 0.45 µm nitrocellulose membrane for further FPLC purification. Unfortunately, enzymatic activity was significantly decreased for wildtype BSLA and it was lost for variant P5F3, suggesting that the BSLA variants were probably not completely soluble after concentration and pH shift, thus purification was not possible using this approach. In order to perform the functional comparison between the parent BSLA and variant P5F3. An SDS-PAGE analysis and relative quantification of the protein bands corresponding to BSLA was performed for both concentrated culture supernatants. Using a lysozyme standard curve (≈14 kDa) as a similarly sized enzyme and gel image analysis using Biorad’ ImageLab software, the enzyme concentration in both samples could be estimated, and thus enzyme concentration for wildtype BSLA (120 ng per reaction) and P5F3 (138 ng per reaction) could be standardized for further enzymatic activity analysis.

**Figure 5:**
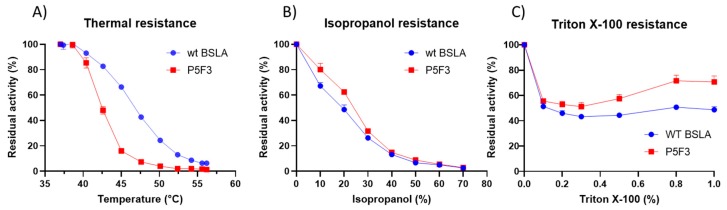
Thermal (**A**), isopropanol (**B**), and Triton X-100 (**C**) resistance for wildtype BSLA and variant P5F3. Thermal resistance for P5F3 is decreased in comparison to WT BSLA, however, isopropanol and Triton X-100 resistance is increased.

Thermal, isopropanol, and Triton X-100 resistance showed similar results as those obtained during screening, whereby P5F3 show a significant decrease in thermal resistance compared to wildtype BSLA, whereas isopropanol and Triton X-100 resistance was increased in the range of 10%–20% and 0.5%–1.0%, respectively, for the deletion/randomization variant (Figure 5). These results confirm that the modifications in region 40–46 of the enzyme, despite having a decreased thermal resistance, showed an increased hydrolytic performance in the presence of isopropanol and Triton X-100.

For a detailed comparison on hydrolytic performance against substrates with different aliphatic chains, saturating curves were performed using *p*-nitrophenyl butyrate (C4), octanoate (C8), dodecanoate (C12), and palmitate (C16) for both enzyme variants. For comparison purposes, the initial rates were fitted to Michaelian curves and “observed” V_max_ and K_m_ values were calculated for each enzyme and substrate series (Table 3).

**Figure A2:**
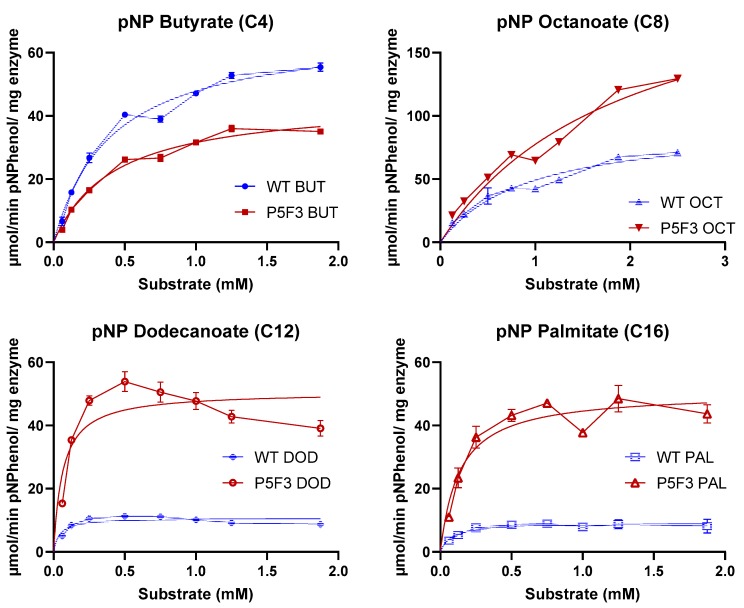
Substrate saturation assays to estimate kinetic parameters of wildtype BSLA and variant P5F3. Enzymatic activity reactions for *p*-nitrophenyl butyrate, octanoate, dodecanoate, and palmitate by measuring the initial rate of reaction (linear range) in microtiter plates as described above at a concentration of 0.06, 0.125, 0.25, 0.5, 0.75, 1.0, 1.25, and 1.875 mM (2.5 mM for pNPO). Initial hydrolysis rates were calculated as µmol of released *p*-nitrophenol per mg of enzyme per minute. For the calculations of the observed enzyme kinetic parameters, the obtained initial rate values for each substrate concentration were used for a Michaelis–Menten fitting using Graph Pad software. V_max_ and K_m_ values obtained for these fittings we used as reference values for comparing the catalytic behavior of both enzyme variants. Activity measurements were performed in triplicate.

The results indicate that both variants have indeed a different behavior for the hydrolysis of the different fatty acid ester substrates. Both enzyme variants exhibited a preference for the octanoate ester substrate (*p*NPO) with the highest observed V_max_ of 94.50 and 229.6 µmol *p*-nitrophenol per mg enzyme per minute for wildtype BSLA and P5F3 variant, respectively, which is in agreement with the original description of the enzyme [45]. Beyond *p*NPO, wildtype BSLA showed a dramatic decrease in hydrolysis performance with longer aliphatic chain substrates *p*NPD and *p*NPP to values in the range of 10 µmol *p*-nitrophenol per mg enzyme per minute, whereas P5F3 maintained its observed V_max_ in the range of 50 µmol *p*-nitrophenol per mg enzyme per minute (Table 3). This experiment allowed to highlight the main difference in the hydrolytic performance between the variants, where P5F3 maintains its performance throughout the assessed substrates, potentially allowing the use of BSLA backbones for the catalysis of longer fatty acid ester substrates. From the substrate saturation curves of the more hydrophobic substrates (Figure A2) for P5F3 variant, we can observe a possible substrate availability (effective concentration) decrease in concentrations higher than 0.5 mM, due to the low water solubility of the substrates and the complex kinetics of substrate availability for lipases [46]. For wildtype BSLA, we can see a significantly lower hydrolytic rate even before 0.5 mM substrate and a saturation beyond that point. This suggests that the substrate affinity, and the ability of the variant to interact with the substrate and perform hydrolysis has been increased, probably also combined with an increased resistance to isopropanol (2.0%–2.5%) and triton X-100 (0.5%–0.63%), both present in the reaction containing *p*NPD and *p*NPP, respectively. Although these experiments were performed using non-purified enzyme preparations, these were standardized for enzyme concentration in the sample, allowing the comparison of the catalytic behavior of the enzyme variants for different fatty acid ester substrates, confirming the differences observed during the screening stage. It must be considered that the reported K_m_ and V_max_ values obtained are not fully accurate as absolute kinetic parameters of these enzymes, and therefore we named them “observed” Km and Vmax values, showing the different performance of the new variant P5F3 with respect to wildtype BSLA. Additionally, it must be considered that substrate availability at high nominal substrate concentrations, especially for *p*NPD and *p*NPP, is probably overestimated, due to limited substrate solubility. Since substrate behavior is the same for both enzyme variants, we propose that the comparison is still valid. Similarly, the calculated values for V_max_ and K_m_ for each substrate used are only presented for comparison purposes, and more precise values should be obtained using a purified enzyme and probably optimized conditions for substrate solubility.

An additional purification strategy for the BSLA variants was attempted by constructing 6x histidine tagged variants by adding the GSSHHHHHHSSG amino acid sequence to the C-terminal end of the enzymes through modification of the *bsla* gene. Though the resulting histidine tagged variants were active, however, their catalytic behavior against fatty acid ester substrates was dramatically modified (Table A2) showing a dramatic decrease of hydrolytic activity against *p*NPD and *p*NPP, thus these variants could not be used as a representation of the unmodified variants. Since BSLA is a relatively small enzyme (181 aa), the introduction of six histidine residues can dramatically affect the ability of the enzyme to interact with substrates having longer hydrophobic fatty acid chains, potentially inhibiting enzyme–substrate interaction. Further modifications and optimization of the histidine tagged version could be achieved by adding a TEV protease target sequence for post purification cleavage of the histidine tag, however, this optimization goes beyond the scope of this work.

In order to investigate if the increased hydrolytic performance is related to a different affinity for the synthetic *p*-nitrophenol group and potentially less related to the fatty acid component of the substrates, the variants were assessed for hydrolysis performance of the fluorogenic 4-methyumbellyferyl-butyrate and -palmitate substrates. For these substrates, the behavior of wildtype BSLA and the variant P5F3 follow the same trend as with the chromogenic *p*-nitrophenyl substrates (Table A3), suggesting that the difference in hydrolytic performance is indeed dependent on the change of the length of the fatty acid esters.

### 2.6. Structure Function Analysis of P5F3

**Figure 6:**
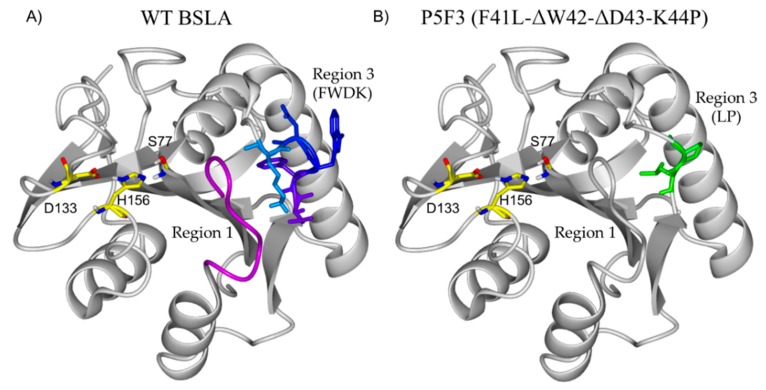
Structural comparison of BSLA (**A**) and the homology model of P5F3 (**B**). Interestingly, region 3 (aa positions 40 to 46) is not in the immediate vicinity of the active site of the enzyme (S77, D133 H156). In fact, region 1 (aa positions 10 to 18, pink) is located closer and between region 3 and the active site. The deleted and substituted amino acids from the original BSLA sequence are FWDR, representing two bulky aromatic residues, a negative and a positive charge residue, with potential functionality and pH response which are replaced by LP in variant P5F3.

The main difference between wildtype BSLA and P5F3 is in the deletion of amino acids in position 42 and 43, and the substitutions Phe41Leu and Arg44Pro therefore, variant P5F3 can be also described as F41L-ΔW42-ΔD43-K44P. To observe the potential changes introduced to P5F3 at the structural level and relate them to the described functional changes, a homology model of the variant was constructed using the Yasara Structure software suite (Figure 6) [42]. Interestingly, region 3 (aa positions 40 to 46) is not in the immediate vicinity of the active site of the enzyme. In fact, region 1 (aa positions 10 to 18) is located closer and between region 3 and the active site. From our screening results, BSLA is strongly sensitive to changes in region 1, since we could not identify active variants for Lib 1, suggesting that this region is either a critical part of the active site, or critical for substrate interaction. The deleted and substituted amino acids from the original BSLA sequence are FWDK (Figure 6A), representing two bulky aromatic residues, a negative and a positive charge residue, with potential functionality and pH response. Kübler et al., through fluorescence spectroscopy analysis of the structural dynamics of BSLA, proposed region 3 as a possible pseudo lid that could affect, through conformational changes, the hydrolytic activity and stability of the enzyme as a response to pH changes (pH 8.5–10) [47]. It would be interesting to perform such analysis for variant P5F3, since in this variant the mentioned loop does not exist and is replaced by a LP amino acid pair (Figure 6B), though the absence of tryptophan 42 could make this study more challenging.

**Figure 7:**
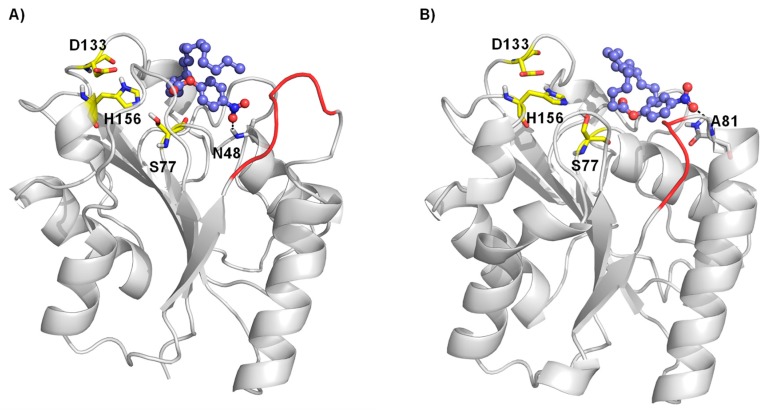
Molecular docking of palmitate substrate in the binding pocket of BSLA WT (**A**) and BSLA P5F3 variant (F41L-ΔW42-ΔD43-K44P) (**B**). Substrate *p*-nitrophenyl palmitate is shown as ball and stick, whereas the catalytic triad (S77, D133 and H156) is shown as yellow sticks. The hydrogen bond interactions are shown with a black dotted line and region 3 is highlighted in red.

To evaluate the potential changes in the preferred interaction between wildtype BSLA and the variant P5F3 a molecular docking experiment was performed using the most common conformation of the BSLA structure and of the constructed homology models from a 100 ns molecular dynamics simulation run. Docking results revealed that in the P5F3 variant, the *p*NPP substrate binds preferentially close to the modified loop 3 region (Figure 7). Palmitate forms a hydrogen bond with N48 in WT (Figure 7A) whereas in P5F3 variant, the hydrogen bond with A81 was observed (Figure 7B). The palmitate substrate binds closer to the catalytic S77 residue, which is involved in the first proton transfer to the substrate for hydrolysis, which could lead to increased activity of the P5F3 variant towards the *p*NPP substrate, compared to WT BSLA.

Secundo et al. performed insertions in region 3 of BSLA, exchanging amino acids 39 to 51 to longer sequences from structural homologues *Fusarium solani pisi* cutinase and *Penicillium purpurogenum acetylxylane* esterase. The resulting variants showed overall decreased hydrolytic activity against tributyrin and pNPP compared to wildtype BSLA, they also showed a modest activity in the transesterification reaction between sulcatol and vinylacetate and were inactive for the alcoholysis of chloro ethyl 2-hydroxy hexanoate with methanol [48]. Our results support the conclusion that region 3 and its modification can modulate the activity of the enzyme and its substrate selectivity. However, contrary to the reported insertions in region 3, our engineering strategy showed that the deletion of two amino acids and double amino acid substitution of the new neighboring residues allowed the generation of a variant with increased hydrolytic activity against longer fatty acid esters and resistance to isopropanol and triton X-100, albeit with a loss in thermal resistance. Additionally, intra-protein hydrogen bond analysis was carried out to attempt to quantify the effect of the amino acid deletion and substitution on the hydrogen bond network and thereby the stability of generated BSLA variant P5F3 compared to BSLA WT. Figure 3 shows that the number of hydrogen bonds in the P5F3 BSLA variant decreases (135 ± 1.94) compared to BSLA WT (141 ± 1.09), this decrease in hydrogen bonds could in part explain the decrease in thermal resistance of the deletion/randomization variant.

The reported catalytic properties of P5F3 show an interesting potential for the development of BSLA variants with modified substrate preference and increased activity for a longer, more hydrophobic fatty acid with aliphatic chains longer than eight carbons [45]. Thus, Variant P5F3 is a potential starting point for further characterization and protein engineering in that direction, including its potential performance in the synthesis reaction in non-aqueous solvents, provided its thermal resistance is also increased through further protein engineering or enzyme immobilization [11].

## 3. Materials and Methods

### 3.1. Reagents and Chemicals

All chemicals and substrates are of analytical grade or higher and were purchased from Sigma–Aldrich (St. Louis, Missouri United States), Merck (Kenilworth, New Jersey, United States) and Cayman Chemical (Ann Arbor, Michigan, United States). Enzymes and dNTPs were obtained from New England BioLabs (Ipswich, Massachusetts, United States). The HPLC-purified oligonucleotides applied for mutagenesis were obtained from Macrogen Inc. (Seoul, Republic of Korea). DNA concentration was quantified using a Biophotometer D30 and the µCuvette accessory (Eppendorf, Hamburg, Germany).

### 3.2. Plasmid Construction and Recombinant Production of BSLA

The amino acid sequence of *Bacillus subtilis* Lipase A (BSLA, PDB 1I6W) was modified with a N-terminal pelB periplasmic destination peptide variant (MGKYLLPTAAAGLLLLAAQPAHM) resulting in a 204 amino acid initial enzyme (MGKYLLPTAAAGLLLLAAQPAHMAEHNPVVMVHGIGGASFNFAGIKSYLVSQGWSRDKLYAVDFWDKTGTNYNNGPVLSRFVQKVLDETGAKKVDIVAHSMGGANTLYYIKNLDGGNKVANVVTLGGANRLTTGKALPGTDPNQKILYTSIYSSADMIVMNYLSRLDGARNVQIHGVGHIGLLYSSQVNSLIKEGLNGGGQNTN). The amino acid numbering for BSLA, however, to facilitate comparison, follows that of the PDB structure (1I6W) assigning number “1” to the 24th amino acid of the construct (Ala) whereas the initial 24 amino acids were numbered from −24 to −1. The resulting peptide was reverse translated into an ORF for *E. coli* expression (atgggcaaatatctgctgccgaccgcagcagcgggtctgctgctgctggcagcacagcctgcacatatggcagaacataatccggttgttatggttcatggtattggtggtgcaagctttaactttgcaggcattaaaagctatctggttagccaaggttggagccgtgataaactgtatgcagttgatttttgggataaaaccggcaccaattataacaatggtccggttctgagccgttttgttcagaaagttctggatgaaaccggtgccaaaaaagttgatattgttgcacatagcatgggtggtgcgaataccctgtattacattaaaaacctggatggtggtaacaaagttgccaatgttgttaccttaggtggtgccaatcgtctgaccaccggtaaagcactgcctggcaccgatccgaatcagaaaattctgtataccagcatttatagcagcgcagatatgatcgtgatgaattatctgagtcgtctggatggcgcacgtaatgttcagattcatggtgtgggtcatattggtctgctgtatagcagccaggttaatagcctgattaaagaaggtctgaacggcggaggtcagaataccaattaa), ordered for synthesis (Macrogen Inc, Seoul, Republic of Korea), and cloned into pET28a(+) using NcoI and EcoRI, generating pET28-pelB-BSLA, used in this work for recombinant production of BSLA. In this system, recombinant protein production is induced by the addition of isopropyl β-D-1-thiogalactopyranoside (IPTG, 0.1–0.5mM) to the culture media or the LB-agar plate.

Recombinant BSLA and the constructed variants were transformed into and produced in *E. coli* BL21 gold (DE3) cells (Agilent, Santa Clara, California, United States). Enzyme production was started by inoculation of an overnight culture in LB-kanamycin medium and adjusting to 0.05 OD_600_, the bacterial culture was then induced using IPTG at ≈1.0 OD_600_ to a final concentration of 0.3 mM. The induced culture was harvested at 16 h post induction and centrifuged (3220*g*, 20 min, 8 °C). The cell culture supernatant was then recovered for further analysis or used directly to measure enzyme activity. Empty vector (pET28a) and non-induced strains were used as negative controls for background signal measurements.

### 3.3. Deletion/Randomization Libraries

Once the target sequences were selected, single stranded DNA oligonucleotide primers were designed to delete two “middle” codons and randomize the new adjacent codons using NNK codons. A modified QuickChange PCR protocol using the plasmid construct of the parental enzyme (pET28-pelB-BSLA) as template [43] was employed for each library generation, using an Aeris Thermal Cycler (Esco, Horsham, PA, United States) and thin-wall PCR tubes (0.2 mL, Bioponte Scientific, Claremont, California, United States). The PCR program used was performed in two steps; in the first reaction, for each library, forward and reverse primers were added in separate tubes, then the following program was performed: 98 °C for 30 s; 98 °C for 10 s, 55 °C for 30 s, 72 °C for 130 s (5 cycles). The forward and reverse reactions of each library were combined, and PCR was continued using the following program: 98 °C for 30s; 98 °C for 10 s, 55 °C for 30 s, 72 °C for 130 s (25 cycles); final elongation 72 °C/10 min. Methylated template DNA was then digested with DpnI (20 U, 37 °C, 18 h). DpnI digested DNA samples were purified and concentrated (DNA Clean and Concentrator Kit, Zymo Research, Irvine, California, United States). The purified DNA was transformed into chemically competent *E. coli* BL21 glod 8DE3) cells and plated in LB-kanamycin agar plater.

### 3.4. Enzyme Production and Library Screening in 96-Well MTP Microtiter Plates

After transformation of the PCR products of the deletion randomization libraries, single transformant colonies were picked and grown on LB/agar/tributyrin/IPTG agar plates, supplemented with kanamycin. After 24 h, the presence of tributyrin clearance halo on each colony was evaluated and compared to the internal controls (empty vector and wildtype colonies) placed in each agar plate. Appropriate variants were picked into a flat bottom 96-well microtiter plate containing 150 µL LB kanamycin medium for master plate generation. Each microtiter plate contained three wells containing wildtype and three empty (no inoculum) wells. Independent of observed hydrolytic in agar plates, at least two plates (180 colonies) were picked for each library. Master plates were incubated for 16 h (37 °C, 500 rpm) in a closed shaker (Jeio Tech shaker, model IST-3075, Seoul, Republic of Korea) having microtiter plated adapters installed. The plates were stored as “master plates” at −80 °C after addition of 100 µL sterile glycerol (50 mL, 50% *w*/*v*) to each well. For recombinant enzyme production of the libraries, each master plate was thawed, replicated into a new 96-well V-bottom microtiter plate containing LB kanamycin medium (150 mL per well) by using a 96-well microtiter plate replicator (own construction, Institute of Biotechnology, RWTH Aachen, Germany) and cultivated for 16 h (37 °C, 500 rpm). The microtiter plates were then replicated into V-bottom, 96-well polystyrene “production” microtiter plates (Corning ref. 3897, Corning, New York, United States) containing 150 µL LB kanamycin medium and placed in the shaker (30 °C, 500 rpm). Turbidity of the wells was monitored using the microtiter plate reader and recombinant enzyme production was induced after the average of OD_600_ of the microtiter plate was 1.0, by adding IPTG to a final concentration of 0.3 mM. BSLA is secreted into the culture supernatant when pET28 is employed in *E. coli* BL21-Gold (DE3) since the N-terminal pelB-signal sequence of the vector enables translocation to the cell periplasm, allowing the accumulation of the recombinant BSLA in the cell culture medium [32]. Therefore, the cell culture supernatant was collected after centrifugation of the expression culture (Eppendorf 5810R, 3220*g*, 20 min, 8 °C) and used for enzyme activity measurements. The coefficient of variation of the OD_600_ when inducing with IPTG was typically 30%. The coefficient of variation of the OD_600_ when harvesting was typically 20%. Using this protocol, the coefficient of variation of the hydrolytic activity against *p*NPB for a plate containing 93 individually picked wildtype BSLA colonies was 18%, which is typically acceptable for screening [49], whereas signal to background was higher than 10-fold.

### 3.5. Esterolytic Activity Measurements Using p-Nitrophenyl Ester Substrates

The esterolytic activity of BSLA was measured in flat bottom polystyrene 96-well microtiter plates (Corning ref. 9017) by the hydrolysis of *p*-nitrophenyl acid esters (butyrate, octanoate, dodecanoate, palmitate) at 37 °C by following the increase of absorbance at 410 nm over time in a microtiter plate reader (Tecan Infinite M200 pro Nano+, Männedorf, Switzerland). The reaction mix was composed of 150 µL of substrate-buffer solution (Tris-HCl pH 7.4 containing 1 mg/ gum arabic, 0.5 mM (unless stated otherwise) *p*-nitrophenyl ester substrate. The reaction was started by adding 15 µL cell culture, concentrated cell culture containing BSLA or purified enzyme solution.

Substrate stock solutions were prepared as follows; *p*-nitrophenyl butyrate, stock 95,6 mM (1g *p*NPB in 50 mL acetonitrile); *p*-nitrophenyl octanoate, stock 75.38 mM (1 g *p*NPO in 50 mL acetonitrile). For *p*NPB and *p*NPO working buffer substrate solutions, Triton X-100 was added for a final concentration of 0.5%.

For *p*-nitrophenyl dodecanoate, a 18.6 mM stock solution was prepared by dissolving 60 mg *p*NPD in 10 mL isopropyl alcohol (80%) and triton X-100 (20%) solution. For *p*-nitrophenyl palmitate, a 15.8 mM stock solution was prepared by dissolving 60 mg *p*NPP in 10 mL isopropyl alcohol (80%) and triton X-100 (20%) solution. The reactions for *p*NPD and *p*NPP contained 2.0% and 2.5% isopropanol, respectively, as well as 0.53% and 0.63% Triton X-100, respectively. All dilutions of stock solutions were prepared fresh before reaction, and *p*NPD and *p*NPP stock solutions and buffers were preincubated at 37 °C before dilution to avoid substrate precipitation. Activity measurements were performed in triplicate.

### 3.6. Thermal Resistance Assay

To asses thermal resistance of BSLA and its deletion/randomization variants, the enzyme was subjected to a fixed temperature incubation, then cooled down and its remaining esterolytic activity was measured. Fifty microliters of BSLA-containing cell culture supernatant were transferred to each well in a 96-well PCR plate (VWR ref. 82006-636) and incubated for 30 min in a temperature ranging from 37 to 57 °C in a temperature gradient thermal cycler (Esco Aeris, Esco, Horsham, PA, United States), and then cooled down to 8 °C. The esterolytic activity of the treated supernatants was measured using the using *p*-nitrophenyl butyrate as substrate. The measured activity at 37 °C was expressed as a percentage of the activity exhibited its corresponding untreated enzyme sample. Each thermal resistance series was performed in triplicate.

### 3.7. Isopropanol and Triton X-100 Resistance Assays

The esterolytic activity of BSLA and its deletion/randomization variants was assessed in the presence of increasing concentrations of isopropanol and of Triton X-100. The microtiter plate based esterolytic assay was performed as described above. Series of working substrate-buffer solutions (0.5 mM *p*NPB) were modified to include 0% to 70% isopropanol, or 0% to 1% Triton X-100. The measured activity at 37 °C was expressed as a percentage of the activity exhibited its corresponding untreated enzyme sample. Each thermal resistance series was performed in triplicate.

### 3.8. Substrate Saturation Assays to Estimate Kinetic Parameters of Wildtype BSLA and Variant P5F3

Enzymatic activity reactions for *p*-nitrophenyl butyrate, octanoate, dodecanoate, and palmitate by measuring the initial rate of reaction (linear range) in microtiter plates as described above at a concentration of 0.06, 0.125, 0.25, 0.5, 0.75, 1.0, 1.25, and 1.875 mM (and 2.5 mM for pNPO). Stock solutions were prepared accordingly to obtain the corresponding final substrate concentrations and maintaining isopropanol and Triton X-100 concentration constant. Initial hydrolysis rates were calculated as µmol of released *p*-nitrophenol per mg of enzyme per minute.

The concentration of *p*-nitrophenol was calculated spectrophotometrically using the Lambert Beer equation using path length 0.58 cm (165 µL in 96-well microtiter plate), and a molar extinction coefficient for *p*-nitrophenol for our system (pH 7.4) of 7194 M^−1^cm^−1^.

**Figure A3:**
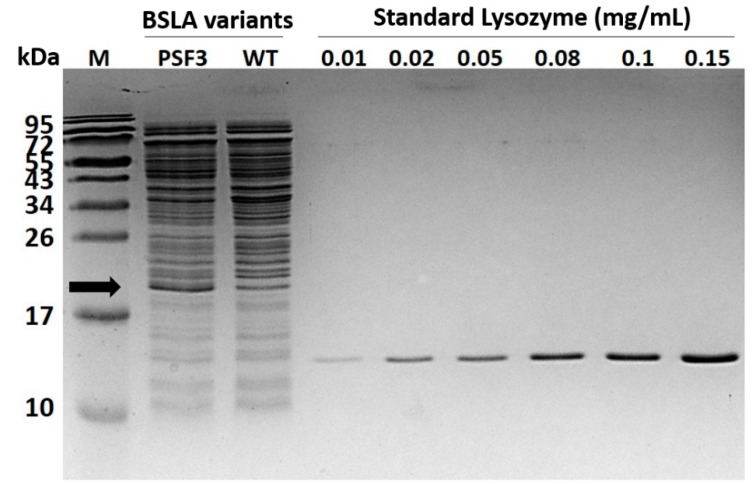
SDS-PAGE of concentrated samples of variant P5F3 and wildtype BSLA. The concentration of BSLA and P5F3 in a 10 kDa mini crossflow concentrated cell supernatant (VivaFlow 200, Sartorius), was estimated through image-based analysis and relative quantitation using a lysozyme calibration curve (Biorad ImageLab). The result was 59.7 µg/mL for P5F3 and 23.0 µg/mL for wildtype BSLA.

The concentration of BSLA and P5F3 in a 10 kDa mini crossflow concentrated cell supernatant (VivaFlow 200, Sartorius), was estimated through image-based analysis and relative quantitation using a lysozyme calibration curve (Biorad ImageLab 6.0.1, Biorad, Hercules, California, United States). After quantification, 120 ng of P5F3 and 138 ng wildtype BSLA were used per reaction (Figure A3).

For the calculations of the observed enzyme kinetic parameters, the obtained initial rate values for each substrate concentration were used for a Michaelis–Menten fitting using Graph Pad software. V_max_ and K_m_ values obtained for these fittings were used as reference values for comparing the catalytic behavior of both enzyme variants. Activity measurements were performed in triplicate.

### 3.9. Esterolytic Activity Measurements Using 4-Methylumbellyferyl Ester Substrates

Microtiter plate enzymatic activity assays were performed using 4-methylumbellyferyl butyrate and palmitate substrates, based on the chromogenic assay described above. Stock solutions of the substrates (2 mM) were prepared in DMSO and diluted to a 50 µM substrate working solution in assay buffer (50 mM Tris-HCl pH 7.4, 1 mg/mL Arabic gum, 0.5 % Triton X-100). The reaction was started by adding 15µL of enzyme or enzyme containing supernatant to 150 µL substrate buffer solution. Substrate hydrolysis and release of fluorescent 4-methylumbelliferone was followed in a microtiter plate reader (Tecan Infinite M200 pro Nano+) using 340 and 450nm as excitation and emission wavelengths, respectively, and the gain setting to 60. The increase in relative fluorescence units over time (RFU/min) in the linear range was used as an indication of substrate hydrolysis. The experiments were performed in triplicate.

### 3.10. Electrophoresis in Polyacrylamide Gels (SDS-PAGE)

Sodium dodecyl sulphate polyacrylamide gel electrophoresis (SDS-PAGE) analysis was performed following the standard method [50], using a 12% acrylamide and a 4% acrylamide stacking gel. Electrophoresis was performed at a fixed current (20 mA per gel).

Concentrated cell culture supernatant (500 µL) was mixed with 300 µL of trichloroacetic acid (TCA) for protein precipitation. After mixing vigorously, the sample was incubated on ice for 30 min. The sample was centrifuged for 10 min at 10,000× *g*, at 8 °C. The supernatant was discarded. The resulting pellet was washed twice using cold acetone. Once the residual acetone was evaporated, the pellet was resuspended in 50 µL of 50 mM Tris buffer pH 8.0 and 15 µL of 4× loading buffer (Biorad) was added. Resuspended samples were incubated for 15 min at 98 °C. Finally, 10 µL of heat-treated sample were loaded in each per well.

### 3.11. Molecular Dynamics Simulation

The chain A of crystal structure of *Bacillus subtilis* lipase A (PDB ID: 1I6W [44]) was used as a starting structure for generation of BSLA variants using YASARA Structure Version 17.6.22 [42] (YASARA Biosciences GmbH, Vienna, Austria). The BSLA P5F3 variant (F41L-ΔW42-ΔD43-K44P) was generated by deleting the amino acid residues in the loop 3 region (41–45 aa) and rejoining it using the loop-remodeling option. The protonation states were assigned based on calculated pKa using PROPKA method and employing the PDB2PQR server [51,52]. Protonation of catalytic triad was determined by considering the catalytic mechanism of lipase [53,54]. The catalytic residues H156 was protonated at Nδ1 atom based the proton transfer mechanism involved in activation of the hydroxyl group of the catalytic serine [53,54,55,56]. The GROMOS96 54a7 force field [57] was used for protein. Hydrogens were added to the protein using pdb2gmx command in GROMACS and the protein molecules were placed in a cubic simulation box (10 nm^3^) and solvated using Single Point Charge (SPC) water model [58,59]. The system was neutralized by adding Cl^-^ ions. The particle mesh Ewald (PME) method [60,61] was applied for electrostatic interactions calculation. Short-range electrostatic interactions and Van der Waals were calculated using a cut-off value 1.0, respectively. Initially, the whole system was minimized using steepest descent minimization algorithm until the maximum force of 1000.0 kJ mol^−1^ nm^−1^ was reached followed by an equilibration under *NVT* and *NPT* ensemble. First, *NVT* equilibration was carried out at constant temperature of 300 K for 100 ps with time step of 2 fs. Second, *NPT* equilibration was carried out at constant temperature of 300 K for 100 ps with time step of 2 fs, respectively. The Berendsen thermostat and Parrinello–Rahman pressure coupling were applied to keep the system at 300 K, time constant (τ_T_) of 0.1 ps and 1 bar pressure, time constant (τ_P_) of 2 ps. Finally, the production run was carried out using *NPT* ensemble for 100 ns with time step of 2 fs at constant temperature of 300 K and coordinates were saved every 200 ps. The LINCS algorithm was used to constraint bonds between hydrogen and heavy atoms. All the calculations were carried out in triplicate using GROMACS 5.1.2 [62,63]. The obtained simulation trajectories were visualized and analyzed using VMD 1.9.1 [64] and GROMACS tools [62,63].

### 3.12. Molecular Docking

The obtained energy minimized structures of WT and the P5F3 BSLA variant were used for molecular docking study of *p*-nitrophenyl palmitate substrate (*p*NPP). The substrate structure was constructed in YASARA and minimized using GAFF with AM1-BCC partial charges employing Ewald particle mesh for long range electrostatic interactions and a direct force cutoff of 10.5 Å. The protein residues were treated with the AMBER ff14 forcefield. A grid box of 12 Å was created around the active site by centering the catalytic triad. Molecular docking of palmitate substrate with BSLA WT and BSLA P5F3 variant was carried out using Autodock 4.2 plug-in within YASARA Structure Version 17.4.17 [42]. A total of 100 docking runs were carried out for each protein backbone and the obtained docking poses were clustered by applying a RMSD cutoff of 0.5 Å and using the default settings available in the YASARA dock_run macro file.

## 4. Conclusions

In this work, we proposed and experimentally performed a novel strategy for enzyme diversity generation, based on the deletion and randomization of non-conserved regions in order to systematically explore the effect of the combination of amino acid substitutions and sequence length variation in enzymes, especially those with already well studied enzyme backbones. Our results also show that the randomization of the neighboring residues is an important feature of the library design in this strategy, because none of the active identified deletion/randomization variants contained a neighbor amino acid pair which would be obtained by simple deletion in the original sequence (GR for Lib 2, FK for Lib 3, RT for Lib 5, and TN for Lib 6, from Table 1) suggesting that, if no randomization of the neighboring joining amino acid residues was performed, no active variant would be found by simply reducing the sequence length.

The generation and identification of BSLA variant P5F3 (F41L-ΔW42-ΔD43-K44P) from Lib 3, having an increased hydrolytic activity against fatty acid esters of long aliphatic chain, supports our claim on the value of applying the deletion/randomization strategy, allowing the rapid identification of novel variants with significant changes in enzymatic activity and offering and alternative path for further research and development on already well studied or highly engineered enzymes which have reached an improvement or performance plateau.

The proposed enzyme engineering strategy also allows for a rapid evaluation of the “sensitivity” of a specific enzyme backbone to sequence and length variation on structurally non-conserved regions, allowing to focus on those that after modification result in active variants with changes in key performance parameters. In the case of the 181 amino acid BSLA, we identified regions 1 and 4 as highly sensitive to deletion/randomization where no active variants were identified, regions 2 and 6 with moderate sensitivity, and region 5 with a low sensitivity where most active variants were identified. Finally, region 3 was identified as a hotspot for functional modulation of the enzyme, where changes in length and sequence can be further studied to generate BSLA variants with increased hydrolytic performance on long chain fatty acid esters.

## Figures and Tables

**Table 1:** Regions in BSLA structure identified through structural alignment and targeted for deletion/randomization. For each region, deleted amino acids are underlined, and randomized pairs are in bold and represented as question marks when randomized.

Identified Region	Library Name	Sequence (Numbers Indicate aa Positions)	Designed Library (aa)
Region 1	Lib 1	10-HGIGGASFN-18	10-HG??SFN-18
Region 2	Lib 2	29-QGWSRD-34	29-Q??D-34
Region 3	Lib 3	40-DFWDKTG-46	40-D??TG-46
Region 4	Lib 4	90-LDGGN-94	90-L??-94-
Region 5	Lib 5	104-GANRLTTGKA-113	104-GAN??GKA-113
Region 6	Lib 6	114-LPGTDPNQKI-123	114-LPG??QKI-123

**Table 2:** Identified amino acid substitutions for active and inactive enzyme variants for each deletion/randomization library of BSLA.

Library	Deleted Positions	Randomized Positions	Randomized Pairs Found in Randomized Positions for Active Variants (Tributyrin-Agar)	Randomized Pairs Found in Randomized Positions for Inactive Variants (Tributyrin-Agar)
Lib 1	13–14	12–15	None found	SE; AT; PR; HL; VV; PI; KG: MK; RS; MG; TQ; DI; TG; PP; LW; QL; NK; SC; AE; IG; VY; TD; GS
Lib 2	31–32	30–33	GY, GI, RG, NL; SR	QY; GF; GR; GT; SY; SL; TY; TI; DW; CC; HA; RW; EP; VL; SP; RC; PA; HT; LS; TP
Lib 3	42–43	41–44	FP; MG; FH; LP; LG; ME; LM	LF; PP; HH; LR; MW; DA; MF; RV; WI; FL; LV; EI; IY; AV
Lib 4	92–93	91–94	None found	KP; CG; QA; IY; RE; VN; LK; MA; GS; SV; LY; DL; LE; AW; TR
Lib 5	108–109	107–110	LG; WG; FG; NG; AG; TG; DG; EG; KG; SG; DG; YG; HG; GG; LV; AP; NA; SA; FP; SP; YS; GS	RC; WD; TH; WF; TA; GE; QA; CC; PF; HV; SC; AQ; TA; PS; GF; PT; RR; KT; PN; NI
Lib 6	118–119	117–120	FI; LH; YM; LY; TQ; LG; PC; KK; RN	LF; PP; HH; LR; MW; DA; MF; RV; WI; FL; LV; EI; IY; AV

**Table 3:** Kinetic parameters observed for wildtype BSLA and variant P5F3 from concentrated cell culture supernatant obtained from Michaelian fitting of the initial rate values resulting from substrate saturation experiments using *p*-nitrophenyl butyrate, octanoate, dodecanoate, and palmitate. Enzyme concentration was estimated by SDS-PAGE analysis and relative quantification of the protein bands corresponding to BSLA using Biorad’ ImageLab software, the enzyme concentration in both samples could be estimated and standardized for each reaction. Michaelis–Menten fitting and kinetic parameter estimation were performed using GrapPad Prism 8.

BSLA	*p*NP Butyrate		*p*NP Octanoate		*p*NP Dodecanate		*p*NP Palmitate	
Variant	Observed V_max_ *	Observed K_m_ (mM)	Observed V_max_ *	Observed K_m_ (mM)	Observed V_max_ *	Observed K_m_ (mM)	Observed V_max_ *	Observed K_m_ (mM)
WT BSLA	67.29 ± 4.64	0.406 ± 0.083	94.50 ± 16.85	0.730 ± 0.342	10.69 ± 0.81	0.042 ± 0.022	9.18 ± 0.77	0.078 ± 0.035
P5F3	44.86 ± 2.34	0.422 ± 0.084	229.6 ± 94.0	1.730 ± 1.296	50.59 ± 4.49	0.064 ± 0.035	50.50 ± 4.15	0.134 ± 0.048

* (µmol *p*-nitrophenol per mg enzyme per min).

**Figure A4:**
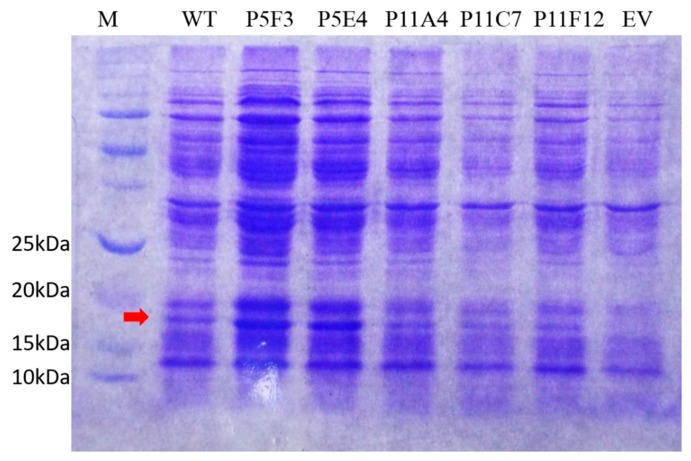
Full SDS-PAGE picture of Figure 3E. (EV = empty vector) the arrow indicates the protein band corresponding to BSLA (≈19 kDa), which is not present in the empty vector (EV) control.

## Appendix A

**Table A1:** Degenerate primers used to generate the deletion/randomization libraries of the *bsla g*ene. Primers pET_T7_HisC_FW and pET_T7_HisC_RV were used to incorporate a C-terminal 6x histidine tag to BSLA and variant P5F3. Randomized nucleotides are underlined.

Primer Name	Library	Sequence
Loop1_lib1_FW	Lib 1.	CCGGTTGTTATGGTTCATGGTNNKNNKAGCTTTAACTTTGCAGGCATTAAAAG
Loop1_lib1_RV	Lib 1	CTTTTAATGCCTGCAAAGTTAAAGCTMNNMNNACCATGAACCATAACAACCGG
Loop2_lib1_FW	Lib 2	GCATTAAAAGCTATCTGGTTAGCCAANNKNNKGATAAACTGTATGCAGTTGATTTTTGGG
Loop2_lib1_RV	Lib 2	CCCAAAAATCAACTGCATACAGTTTATCMNNMNNTTGGCTAACCAGATAGCTTTTAATGC
Loop3_lib1_FW	Lib 3	CCGTGATAAACTGTATGCAGTTGATNNKNNKACCGGCACCAATTATAACAATGG
Loop3_lib1_RV	Lib 3	CCATTGTTATAATTGGTGCCGGTMNNMNNATCAACTGCATACAGTTTATCACGG
Loop4_lib1_FW	Lib 4	CGAATACCCTGTATTACATTAAAAACCTGNNKNNKAAAGTTGCCAATGTTGTTACCTTAG
Loop4_lib1_RV	Lib 4	CTAAGGTAACAACATTGGCAACTTTMNNMNNCAGGTTTTTAATGTAATACAGGGTATTCG
Loop5_lib1_FW	Lib 5	GTTACCTTAGGTGGTGCCAATNNKNNKGGTAAAGCACTGCCTGGC
Loop5_lib1_RV	Lib 5	GCCAGGCAGTGCTTTACCMNNMNNATTGGCACCACCTAAGGTAAC
Loop6_lib1_FW	Lib 6	GGTAAAGCACTGCCTGGCNNKNNKCAGAAAATTCTGTATACCAGCATTTATAGCAG
Loop6_lib1_RV	Lib 6	CTGCTATAAATGCTGGTATACAGAATTTTCTGMNNMNNGCCAGGCAGTGCTTTACC
pET_T7_HisC_FW	Histag	TAAGAGAATTCGAGCTCCGTCG
pET_T7_HisC_RV	Histag	CTCGAATTCTCTTATTAGCCGCTGCTGTGATGATGATGATGATGGCTGCTGCCATTGGTATTCTGACCTCCGCC

**Table A2:** Activity comparison for wildtype BSLA and P5F3 with their corresponding variants containing a C-terminal 6x histidine tag.

	µmol *p*-Nitrophenol Per mg Enzyme Per min (0.5 mM Substrate)			
	*p*NP-Butyrate	*p*NP-Octanoate	*p*NP-Dodecanoate	*p*NP-Palmitate
WT	42.51 ± 2.76	50.88 ± 3.31	48.99 ± 4.16	28.56 ± 1.71
WT Histag	40.35 ± 3.03	40.10 ± 2.57	17.54 ± 1.35	9.24 ± 0.63
P5F3	44.13 ± 3	53.58 ± 3.70	53.95 ± 3.94	48.27 ± 3.62
P5F3 Histag	41.47 ± 3.32	51.64 ± 3.87	35.21 ± 2.29	21.30 ± 1.38

**Table A3:** Activity comparison for wildtype BSLA and P5F3variant using the fluorogenic substrate 4-methyl umbelliferyl-fatty acid ester (BUT or PAL) instead of the chromogenic *p*-nitrophenyl fatty acid ester substrates.

	RFU/min (4-Methyl Umbelliferyl-BUT or PAL)		
Enzyme	BUT	PAL	Ratio PAL/BUT
WT	487 ± 21	208 ± 41	0.4
P5F3	483 ± 36	381 ± 26	0.8
No enzyme	1	−1	
